# Pregnancy risk and contraception among reproductive-age women with rheumatic heart disease attending care at a tertiary cardiac center in Tanzania: a hospital-based cross-sectional study

**DOI:** 10.1186/s12905-023-02332-0

**Published:** 2023-08-31

**Authors:** David G. Paulo, Reuben Mutagaywa, Henry Mayala, Aileen Barongo

**Affiliations:** 1 Department of Internal Medicine, School of Clinical Medicine, Muhimbili University of Health and Allied Sciences, Dar Es Salaam, Tanzania; 2 Jakaya Kikwete Cardiac Institute, Dar Es Salaam, Tanzania; 3 Mwananyamala Regional Referral Hospital, Dar Es Salaam, Tanzania

**Keywords:** Pregnancy risk, Contraception, Reproductive-age women, Rheumatic heart disease

## Abstract

**Background:**

Rheumatic heart disease (RHD) remains prevalent in the developing world and reproductive-age women are disproportionately affected. It is among the common est cardiac diseases during pregnancy and is associated with poor pregnancy outcomes. Despite its importance among reproductive-age women, there are no local studies that characterize the clinical characteristics, risk of poor pregnancy outcomes and contraception which represents one effective way to prevent unplanned pregnancies in this population.

**Methods:**

This was a hospital-based descriptive cross-sectional study. Non-pregnant reproductive-age women with echocardiographically diagnosed RHD were consecutively recruited from in- and out-patients units of the Jakaya Kikwete Cardiac Institute (JKCI). A clinical research form was used to gather socio-demographic, clinical characteristics, contraception status and echocardiographic information. The maternal/pregnancy risk class was determined using the modified World Health Organization (WHO) classification of maternal risk.

**Results:**

Two hundred thirty-eight women of reproductive age with RHD were recruited. The median age (range) was 36 years (15–49). Two-thirds were dyspneic on moderate exertion and 17.2% had New York Heart Association class IV heart failure. A quarter had atrial fibrillation/flutter. On echocardiography, mitral regurgitation was the most common valvular lesion (68.1%), followed by mitral stenosis (66.8%), and 12.2% of participants had reduced left ventricular ejection fraction. Two-thirds (66%) had a high pregnancy risk (class IV) based on the modified WHO classification system. The proportion of participants using contraception was 7.1% and common methods were: bilateral tubal ligation 5 of 17 (29.4%) and hormonal implant (4 of 17). The most common reason for the choice of a method was safety, 10 out of 17 (58.8%).

**Conclusion:**

The majority of women of reproductive age with RHD in our hospital cohort are at the highest pregnancy risk based on the modified WHO classification and a very small proportion of them are on contraception. These results call for action among clinicians to offer counselling to these patients, educating them on their risk and offering appropriate contraception advice while waiting for definitive interventions.

## Background

While rheumatic heart disease (RHD) is currently rare in the developed world, low and middle income countries are still heavily affected [1] with females being more commonly affected contributing almost two- thirds of all patients with RHD [2].

In South Africa, RHD contributes up to 30% of heart diseases during pregnancy [3]. A recent study in Kenya found a 5 per 1000 prevalence of RHD among low risk pregnancies [4]. Rheumatic heart disease is associated with poor pregnancy outcomes including prematurity, low birth weight, worsenig heart failure, arrythmias and maternal mortality [5, 6]. This can partly be explained by the fact that pregnancy is a high output state characterised by an increase in effective circulating volume, heart rate, a decrease in peripheral resistance and apparent anemia due to hemodilution [7–10] and these physiological changes are poorly tolerated by women with pre-existing heart disease including RHD.

The 2018 European Society of Cardiology (ESC) guidelines on the management of cardiovascular diseases during pregnancy recommend performing risk assessment in all women of childbearing age with cardiac diseases using the modified WHO classification of maternal risk [11]. In this classification system, there are five classes of cardiac diagnoses: I, II, II-III, III and IV. Diagnoses included in class IV include: severe mitral stenosis, severe symptomatic aortic stenosis, severe left ventricular systolic dysfunction (ejection fraction less than 30%) among others. The higher the class, the more the risk of complications. Pregnancy is contraindicated in patients who fall in class IV. Hence counselling is recommended in all women of reproductive age with heart disease including RHD prior to conception including offering contraception to prevent unplanned pregnancies.

Contraception represents one of the effective ways to reduce the risk of unplanned pregnancies and hence poor pregnancy outcomes among reproductive age women with RHD. However, the very few studies conducted in this group have revealed a disappointingly low uptake of contraception [2, 12].

Despite the large burden of RHD among reproductive age women in Sub- Saharan Africa, there is regional scarcity as well as total absence of local systematically collected data on the maternal risk using the modified WHO classification, and proportion of contraception in this patient population. This study was set out to fill this gap by collecting data on the clinical characteristics, echocardiographic findings, pregnancy risk and contraception among reproductive age women with RHD. Reliable data on pregnancy risk and contraception among reproductive age women with RHD will help clinicians to come up with strategies to improve patient care and ensure better pregnancy outcomes. Data from this study may be instrumental in influencing development of local guidelines on management of reproductive age women with RHD. Maternal mortality rates are still comparatively high in the developing world and heart diseases including RHD are contributory. Data from this study will pave a way for more research and intervention among reproductive age women with RHD with respect to reproductive health and hence contribute to reduction in maternal mortality.

## Methods

This was a hospital-based cross-sectional study conducted from August, 2021 to January, 2022 in inpatient wards and outpatient clinics of the Jakaya Kikwete Cardiac Institute (JKCI) which is a national tertiary cardiac care center located in Dar es Salaam, Tanzania. It receives cardiac patients from all across the country for both medical and surgical management. It serves more than 800 patients per week.

### Study participants

The study population was women of reproductive age (15–49 years) with an echocardiographic diagnosis of RHD according to the World Heart Federation criteria [13]. The calculated sample size was 238 using the single population proportion formula assuming 95% confidence interval, 5% marginal error 19% proportion from a previous study in Myanmar [12] which found a 19% proportion of contraception among reproductive age women with RHD. A consecutive recruitment method was used to obtain the required sample size. Pregnant women,those on whom surgical correction of the RHD lesions was performed and those who underwent valvulopasty were excluded. No women were excluded based on the severity of valve lesions and none declined to participate in the study.

### Operational definitions

Pregnancy risk in this study refers to the risk of adverse pregnancy outcomes due heart disease as per the modified WHO risk classification.

Reproductive age women refer to women in the age range of 15 to 49 years.

Contraception in this study is defined as the use of family planning methods like hormonal implants, contraceptive pills, barrier methods, hormonal injections, intrauterine devices, sterilization. Calendar method was not included in this study.

### Study procedures

The investigators visited the cardiac wards, cardiac clinics and the echocardiography room on a daily basis to look for reproductive age women with an echocardiographic diagnosis of RHD. Once these were identified, they were informed and asked to participate in the study. All participants were assured that participation in the study was voluntary and that there are no negative consequences for those who decline to participate. All patients underwent physical examination and the findings of Jugular Venous Pressure, edema, lung crepitations were recorded. Framingham criteria were used to make a clinical diagnosis of heart failure. Patients were then classified to New York Heart Association (NYHA) classes I – IV according to their symptoms. A 12-lead resting electrocardiogram was obtained from all patients. A Bionet Cardio 7 machine was used. Reading of the ECGs was done manually by the first author (Internal Medicine specialist) and reviewed by the second author who is a senior cardiologist.

Echocardiography was performed on each participant to record the kinds of valvular lesions and their severity in terms of valve area or regurgitation, the left ventricular ejection fraction and chamber sizes. The echocardiogram examinations followed the American Society of Echocardiography guidelines [14, 15]. An SC 2000 Siemens echocardiography machine was used. The collected clinical and echocardiographic data were used to classify the pregnancy risk of reproductive age women with RHD using the modified WHO pregnancy risk classification system as shown in Table 1.

**Table 1:** Modified WHO classification of maternal cardiovascular risk (only part relevant to study included)

**II-III (intermediate increased risk of maternal mortality or moderate to severe increase in morbidity)**	**III (significantly increased risk of maternal mortality or severe morbidity)**	**IV (extremely high risk of maternal mortality or severe morbidity)**
Mild left ventricular impairment	Moderate left ventricular impairment (ejection fraction 30–45%)	Severe systemic ventricular dysfunction (EF < 30% or NYHA III-IV)
Valve disease not considered I or IV (mild mitral stenosis, moderate aortic stenosis)	Moderate mitral stenosis (1–1.5cm^2^)	Severe mitral stenosis (< 1cm^2^)
	Severe asymptomatic aortic stenosis (< 1cm^2^)	Severe symptomatic aortic stenosis (< 1cm^2^)

Using a structured questionnaire, participants were asked if they were currently on any method of contraception and if yes what type of contraception and the reasons for their choice of the specific type of contraception. This information was recorded. Calendar method of contraception was not included in this study. A clinical research form was used to record the demographic characteristics and clinical data.

### Data analysis

The collected data was checked for quality and coding was done prior to entering into the computer statistical program. Data was analyzed using SPSS for Windows version 23. Data was presented as mean ± SD/ median with range for continuous variables and as percentages for categorical variables.

### Ethical consideration

This study received ethical clearance from the institutional review board of the Muhimbili University of Health and Allied Sciences. Informed consent/ascent was obtained from all participants.

## Results

A total of 238 reproductive age women with echocardiographically diagnosed RHD participated in the study. The median age (range) of the participants was 36 years (15–49). Sixty-five percent were married. More than three quarters (*n* = 195/238) of the study participants were seen at the outpatient clinic and 18.1% were admitted. Almost two-thirds (*n* = 150/238) reported to be dyspneic on moderate exertion. More than two-thirds were in NYHA class II and 17.2% were in class IV heart failure. Majority had a normal ECG while a quarter had atrial fibrillation/flutter (Table 2).

**Table 2:** Socio-demographic and clinical characterisitics of participants (*N* = 238)

Variable	Frequency
Median age (Range)	36 (15–49)
**Age groups, n (%)**	
15–19	14 (5.9)
20–34	90 (37.8)
35–49	134 (56.3)
**Marital status, n (%)**	
Married	155 (65.1)
**Education level, n (%)**	
Secondary	102 (42.9)
**Visit type, n (%)**	
Outpatient	195 (81.9)
**Clinical findings, n (%)**	
Dyspnea on exertion	150 (63.0)
NYHA class IV heart failure	41 (17.2)
Atrial fibrillation on ECG	64 (26.9)

### Echocardiographic findings

The most common valvular lesion was mitral regurgitation (MR) followed by mitral stenosis (MS). Left atrial dilatation was present in more than a quarter and 12.2% had reduced left ventricular ejection fraction (Table 3).

**Table 3:** Echocardiographic findings of participants (*N* = 238)

Variable	Frequency
**Valve lesions, n (%)**	
Mitral regurgitation^a^	162 (68.1)
Moderate	38 (16.0)
Severe	85 (35.7)
Mitral stenosis^a^	159 (66.8)
Moderate	30 (12.6)
Severe	121 (50.8)
Aortic regurgitation	88 (37)
Aortic stenosis^a^	14 (5.9)
Severe	7 (2.9)
Tricuspid regurgitation	103 (43.3)
**Cardiac chambers, n (%)**	
Left ventricular hypertrophy	10 (4.2)
Left ventricular dilatation	57 (23.9)
Right ventricular dilatation	19 (8.0)
Left atrial dilatation	186 (78.2)
Right atrial dilatation	79 (33.2)
**Left ventricular ejection fraction, n (%)**	
Normal (≥ 50%)	174 (73.1)
Mildly reduced (41–49%)	35 (14.7)
Reduced (≤ 40%)	29 (12.2)

^a^some categories of severity (mild, moderate or severe) are not included to reduce congestion in the table

### Pregnancy risk of the participants

**Fig. 1:**
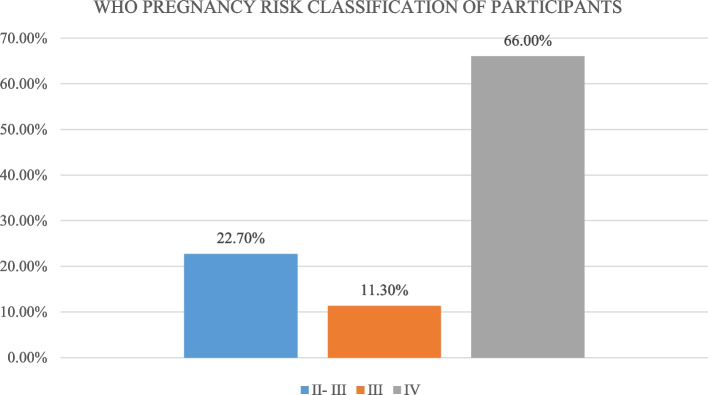
Pregnancy Risk Classification Of Participants

Based on the modified WHO maternal risk classification system for women of reproductive age with heart disease, 66% of the participants were in class IV (Fig. 1).

### Contraception among participants

The proportion of reproductive age women with RHD who were using contraception was 7.1%. In terms of age groups: none of those in the 15–19 age group was using contraception while 7.8% (7 of 90) and 7.5% (10 of 134) of the 20–34 and 35–49 age groups were using contraception respectively. Married women comprised 13 of the 17 women using contraception. Only 11 of 157 (7%) women on modified WHO class IV were using contraception. The most commonly used contraceptive method was bilateral tubal ligation (29.4%), followed by hormonal implant (4 of 17). The most common reason for choice of a particular method was safety (58.8%) (Table 4).

**Table 4:** Contraceptive methods used by participants and reasons for their choice (*N* = 17)

Variable	Frequency
**Contraceptive method, n (%)**	
Hormonal implant	4 (23.5)
Intrauterine contraceptive device	3 (17.7)
Combined oral contraceptives	2 (11.8)
Barrier method	1 (5.9)
Hormonal injection	2 (11.8)
Bilateral tubal ligation	5 (29.4)
**Reasons for choice, n (%)**	
Safe	10 (58.8)
Easy	4 (23.5)
Reversible	2 (11.8)
Advised by health worker	1 (5.9)

## Discussion

This study found out that a large proportion (66%) of reproductive age women with RHD at our center are at the highest risk (class IV) of poor pregnancy outcomes based on the modified WHO pregnancy risk classification system. It also found out that the proportion of these women on contraception is very low (7.1%).

This is the first local study to report maternal risk among reproductive age women with RHD. There are no local and regional studies that have categorized reproductive age women with RHD based on this risk classification system and hence this study serves as an eye opener that most women with RHD carry the highest maternal risk. Approximately two-thirds of the study participants had the highest maternal risk (class IV) based on the modified WHO maternal risk classification system. The clinical and echocardiographic findings contributing to this high risk include severe MS, severe symptomatic aortic stenosis (AS), class III or IV heart failure and a reduced ejection fraction < 30%. This represents a patient population in whom pregnancy is contraindicated and if they present pregnant, termination should be considered. In agreement with the current study, a study conducted in India found Class IV WHO risk category to be the most predominant among 80 pregnant women with native valve RHD or after valve intervention [16]. In this study, 36.2% pregnant women had modified WHO risk score class IV. The differences in the proportions between the two studies are due to the fact that, in the Indian study, majority of the women (63.7%) had undergone prior intervention either balloon valvotomy or valve replacement which downgrades the risk from class IV to III.

Despite the observed high maternal risk in this study, the proportion of reproductive age women with RHD who were on contraception in this study were only 7.1%. In line with these findings, The REMEDY study (conducted among patients with RHD in 12 African countries, India and Yemen and involving 1825 reproductive age women) found an even lower proportion of contraception at 3.6% among women aged 12–51 years [2]. A study conducted in Myanmar found that in the same population of reproductive age women with RHD, the proportion of contraception was 19% [12]. Overall, very few studies have investigated contraception among reproductive age women with RHD. Cardiology authorities, both regional [17] and global [11] advocate counselling to these patients including offering contraception. In the current study, almost half of all participants were told by their attending doctor that they were not strong enough to be pregnant but they were not specifically told to be on contraception. A study done in New Zealand found that contraception was discussed with only 38% of patients with heart disease and it was less likely to be discussed with RHD patients [18]. The reasons could be a high workload leading to shortage of time to discuss contraception options, lack of contraception expertise among clinicians attending these patients and cultural issues.

The most common method of contraception was bilateral tubal ligation used by 5 of the 17 participants who were on contraception followed by hormonal implant used by 4 participants. An intrauterine device was used by 3 participants. In this patient population, the type of contraceptive method used is also important both in terms of efficacy and safety. One participant was using barrier method. The failure rate of this method is high and hence for a reproductive age woman with high maternal risk RHD, it is not an appropriate alternative for prevention of pregnancy. Combined oral contraceptives are prone to drug- drug interactions and carry a pro-thrombotic risk and hence may not be a good alternative among most in this patient population especially those on warfarin (44.1% in this study), left atrial enlargement and a previous history of thromboembolism. This is another area of action where by clinicians should counsel patients on the pros and cons of the different methods but in the context of their heart disease and pertinent issues like drug interactions and efficacy of a particular method.

The most common reason for choice of a particular contraception method was safety (58.8%) followed by ease of use and reversibility. These are important findings, as they will help in encouraging and supporting reproductive age women with RHD to make informed decisions based on contraception advice. The issue of reversibility is particularly important because one study has shown that despite their heart disease, these women are still wishing to conceive [19]. In addition to that, reproductive decisions in our setting are still highly dependent on the male spouse and hence these factors should be taken into consideration when counselling for contraception among high risk reproductive age women with RHD.

This study was conducted at a tertiary level health facility which is the only center in the country that offers surgical and minimally invasive procedures for valvular abnormalities in RHD. Hence the preponderance of high risk valvular lesions like MS might represent a selected patient population awaiting intervention and hence not a true representation of the picture in the general population. However, the study still provides valuable evidence that high risk lesions are prevalent among reproductive age women with RHD and hence holistic care including addressing reproductive health issues should be put into consideration.

## Conclusion

The Majority of reproductive age women with RHD in this study cohort were found to be at the highest pregnancy risk based on the modified WHO classification and a very small proportion of them are on contraception. These results call for action among clinicians to offer counselling to these patients, educating them on their risk and offering appropriate contraception advice while waiting for definitive interventions. Further research is also warranted in order to establish patient and clinician associated factors that affect contraception in this patient population.

## Data Availability

The datasets generated and analyzed during the current study are available from the corresponding author on reasonable request.

## Abbreviations

ECG: Electrocardiography
JKCI: Jakaya Kikwete Cardiac Institute
MR: Mitral regurgitation
MS: Mitral stenosis
NYHA: New York Heart Association
RHD: Rheumatic heart disease
WHO: World Health Organization

## References

[1] Antunes MJ (2020). The global burden of rheumatic heart disease: Population-related differences (it is not all the same!). Brazilian J Cardiovasc Surg.

[2] Zühlke L, Engel ME, Karthikeyan G, Rangarajan S, Mackie P, Cupido B (2015). Characteristics, complications, and gaps in evidence-based interventions in rheumatic heart disease: The global rheumatic heart disease registry (the REMEDY study). Eur Heart J.

[3] Sliwa K, Libhaber E, Elliott C, Momberg Z, Osman A, Zühlke L (2014). Spectrum of cardiac disease in maternity in a low-resource cohort in South Africa. Heart.

[4] Snelgrove JW, Alera JM, Foster MC, Kipchumba CN, Bloomfield GS, Silversides CK (2021). Prevalence of rheumatic heart disease and other cardiac conditions in low-risk pregnancies in kenya : a prospective echocardiography screening study. Glob Heart.

[5] Diao M, Kane A, Ndiaye MB, Mbaye A, Bodian M, Dia MM (2011). Pregnancy in women with heart disease in sub-Saharan Africa la grossesse des femmes atteintes de cardiopathie en Afrique subsaharienne. Arch Cardiovasc Dis.

[6] Roos-Hesselink J, Baris L, Johnson M, De Backer J, Otto C, Marelli A (2019). Pregnancy outcomes in women with cardiovascular disease: Evolving trends over 10 years in the ESC Registry of Pregnancy and Cardiac disease (ROPAC). Eur Heart J.

[7] Adam K (2017). Pregnancy in Women with Cardiovascular Diseases. Methodist Debakey Cardiovasc J.

[8] Thorne SA (2004). Pregnancy in heart disease. Heart.

[9] Anthony J, Osman A, Sani MU (2016). Valvular heart disease in pregnancy. Cardiovasc J Afr.

[10] Stout KK, Otto CM (2007). Pregnancy in women with valvular heart disease. Heart.

[11] Regitz-Zagrosek V, Roos-Hesselink JW, Bauersachs J, Blomström-Lundqvist C, Cífková R, De Bonis M (2018). ESC Guidelines for the management of cardiovascular diseases during pregnancy. Vol. 39. Eur Heart J.

[12] Myint NPST, Aung NM, Win MS, Htut TY, Ralph AP, Cooper DA (2018). The clinical characteristics of adults with rheumatic heart disease in Yangon, Myanmar: An observational study. PLoS ONE.

[13] Reményi B, Wilson N, Steer A, Ferreira B, Kado J, Kumar K (2012). World Heart Federation criteria for echocardiographic diagnosis of rheumatic heart disease—an evidence-based guideline. Nat Rev Cardiol.

[14] Baumgartner H, Hung J, Bermejo J, Chambers JB, Evangelista A, Griffin BP, Iung B, Otto CM, Pellikka PA, Quiñones M; American Society of Echocardiography; European Association of Echocardiography. Echocardiographic assessment of valve stenosis: EAE/ASE recommendations for clinical practice. J Am Soc Echocardiogr. 2009;22(1):1-23. quiz 101-2. 10.1016/j.echo.2008.11.029. Erratum in: J Am Soc Echocardiogr. 2009;22(5):442. Erratum in: J Am Soc Echocardiogr. 2023;36(4):445.

[15] Lang RM, Badano LP, Mor-avi V, Afilalo J, Armstrong A, Ernande L (2015). Recommendations for cardiac chamber quantification by echocardiography in adults : an update from the American society of echocardiography and the European association of cardiovascular imaging. J Am Soc Echocardiogr.

[16] Khanna R, Chandra D, Yadav S, Sahu A, Singh N, Kumar S (2021). Maternal and fetal outcomes in pregnant females with rheumatic heart disease. Indian Heart J.

[17] Mocumbi AO, Jamal KKF, Mbakwem A, Shung-King M, Sliwa K (2018). The Pan-African society of cardiology position paper on reproductive healthcare for women with rheumatic heart disease. Cardiovasc J Afr.

[18] Dalen CT, Fairley SL, Aitken A, Yan W, Li G (2021). Contraception and pre-conception counselling in cardiac patients : we can do better experience from a tertiary centre in New Zealand. Hear Lung Circ.

[19] Chang AY, Nabbaale J, Nalubwama H, Okello E, Ssinabulya I, Longenecker CT (2018). Motivations of women in Uganda living with rheumatic heart disease: A mixed methods study of experiences in stigma, childbearing, anticoagulation, and contraception. PLoS ONE.

